# Eyelid Dirofilaria During COVID-19 Pandemic: A Telemedicine Diagnosis

**DOI:** 10.7759/cureus.15525

**Published:** 2021-06-08

**Authors:** Sahil Agrawal, Sujeeth Modaboyina, Nimmy Raj, Deepsekhar Das, Mandeep S Bajaj

**Affiliations:** 1 Ophthalmology, All India Institute of Medical Sciences, New Delhi, IND

**Keywords:** telemedicine, dirofilaria, parasite infection, eyelid mass, subcutaneous dirofilaria

## Abstract

Telemedicine is an important tool improving the delivery of health care services for diagnosis, treatment and prevention of diseases. A 22-year-old female consulted over video teleconsultation for a swelling in the left upper eyelid, which also moved at certain times for eight days. The video revealed a vermiform swelling on her left upper eyelid with occasional movements. A provisional diagnosis of subcutaneous eyelid Dirofilaria infection was made, and with the help of a surgeon practising locally, it was removed in total. The worm was 40 mm in length and had a slender white body, identified as a sexually immature female Dirofilaria repens. Herein, we share this exciting experience of diagnosing subcutaneous eyelid dirofilariasis at a telemedicine video conference.

## Introduction

Amidst the pandemic, telemedicine has become an essential and reliable platform for screening patients and reducing exposure to COVID-19. Herein, we share an exciting experience of diagnosing a subcutaneous eyelid dirofilariasis through a telemedicine video conference for a patient located 2,700 kilometres across the nation. 

## Case presentation

**Figure 1:**
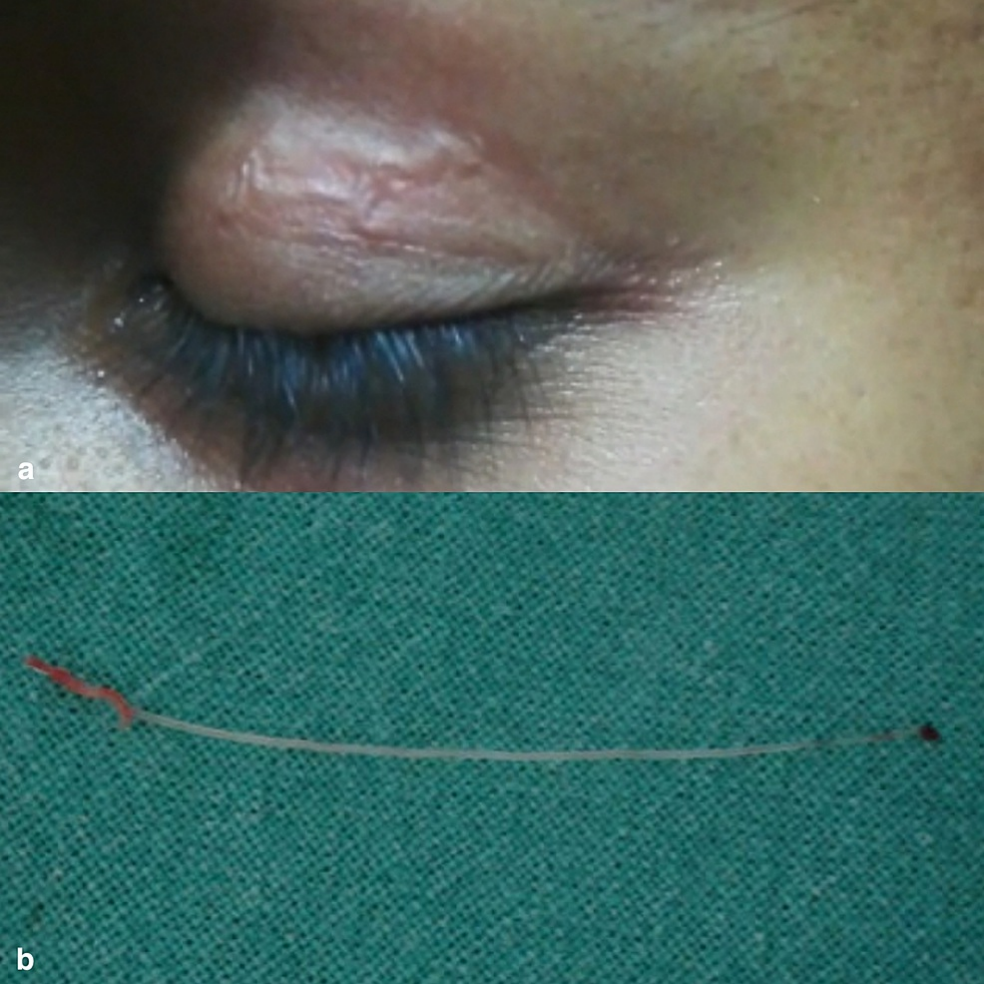
(a) Clinical picture showing a vermiform swelling in the upper lid. (b) Clinical picture of the surgically removed nematode.

A 22-year-old female consulted us by telemedicine during the COVID-19 pandemic lockdown; her primary complaint was a swelling in her left upper eyelid that she had noticed the previous day. She mentioned that the swelling also moved at certain times. She sent a picture and a video during the telemedicine conference, documenting the same (Figure 1a, Video 1).

 

**Video 1:** Smartphone video sent by the patient showing a vermiform swelling in the upper lid with occasional movement.

The video revealed a vermiform swelling on her left upper eyelid with occasional movements. The patient had no other ocular or systemic symptoms. A provisional diagnosis of subcutaneous eyelid Dirofilaria infection was made. She was asked to visit the hospital for the removal of the parasite and systemic evaluation. Meanwhile, her complete haemogram was within normal limits.

On reporting to the hospital, the patient was examined thoroughly for any other site with similar lesions, but none was found. On ophthalmological examination, except for the eyelid swelling, her eyes were otherwise normal. An examination of the patient’s stool did not reveal any parasite ova or cysts, and peripheral blood did not show eosinophilia. The worm was surgically removed from the eyelid using forceps. The worm was 40 mm in length and had a slender white body (Figure 1b). It was identified to be a sexually immature female Dirofilaria repens by the microbiology department.

## Discussion

Dirofilariasis is usually seen in wild animals. Various species of Dirofilaria, including D. immitis, D. repens, D. striata, D. tenuis, D. Ursi, and D. spectans, have been reported to cause incidental infections in humans [1]. The Aedes, Armigeres, Culex, Anopheles, and Mansonia species of mosquitoes are involved in the transmission of this parasite [1]. Although this zoonosis is endemic in Mediterranean countries, there are reports from Africa, Asia, America, and Australia as well [2].

Dirofilaria and Nochitella are two subgeneras of the genus Dirofilaria. Nematodes belonging to the subgenus Dirofilaria have cuticles that are smooth and usually affect the pulmonary arteries. The nematodes of the Nochitella subgenus have longitudinal cuticular ridges and are located in subcutaneous tissues [3]. D. repens belongs to the subgenus Nochitella. There are multiple reports of it affecting the subcutaneous spaces of the forearm, axilla, and breast [1,4]. Ocular infection is rare; however, subconjunctival, eyelid, orbital, and even intraocular cases have been reported [3].

 Surgical removal is the treatment of choice, and diagnosis is confirmed by histopathological examination [1,2]. In rare cases, blood eosinophilia or an elevated serum immunoglobulin E (IgE) level may be noted, which limits the use of eosinophilic counts and serum IgE level in screening for dirofilariasis in suspected individuals. A deoxyribonucleic acid (DNA) extraction along with a pan-filarial polymerase chain reaction (PCR) can be used to confirm the diagnosis of D. repens [1,2].

The patient on retrospectively enquiring stated that she worked as a farmer and was quite disturbed mentally with the unsightful appearance of the lesion. She was quite relieved after the surgery. This case highlights the importance of telemedicine, which can be used as a safe screening tool for detecting diseases and determining whether a patient needs to visit the hospital.

## Conclusions

Ocular dirofilariasis has increased in recent years with climatic change mainly in warm and moist areas. D. repens is responsible for ocular dirofilariasis and the only appropriate management is the removal of the parasite. Adequate measures like mosquito control in such regions should be performed to prevent such incidences. This case highlights the importance of telemedicine, which can be used as a safe screening tool for detecting diseases and determining whether the patient needs to visit the hospital.

## References

[1] Yaranal PJ, Priyadarshini MM, Purushotham B (2015). Human subcutaneous dirofilariasis of forearm an unusual presentation. Indian J Dermatol.

[2] Kalogeropoulos CD, Stefaniotou MI, Gorgoli KE, Papadopoulou CV, Pappa CN, Paschidis CA (2014). Ocular dirofilariasis: a case series of 8 patients. Middle East Afr J Ophthalmol.

[3] Joseph E, Matthai A, Abraham LK, Thomas S (2011). Subcutaneous human dirofilariasis. J Parasit Dis.

[4] Maltezos ES, Sivridis EL, Giatromanolaki AN, Simopoulos CE (2002). Human subcutaneous dirofilariasis: a report of three cases manifesting as breast or axillary nodules. Scott Med J.

